# Farmer and Veterinary Practices and Opinions Related to the Diagnosis of Mastitis and Metabolic Disease in UK Dairy Cows

**DOI:** 10.3389/fvets.2020.00127

**Published:** 2020-03-04

**Authors:** Francesc X. Donadeu, Natalie L. Howes, Cristina L. Esteves, Martin P. Howes, Tim J. Byrne, Alastair I. Macrae

**Affiliations:** ^1^The Roslin Institute and Royal (Dick) School of Veterinary Studies, University of Edinburgh, Easter Bush, Midlothian, United Kingdom; ^2^AbacusBio International Ltd., Easter Bush, Midlothian, United Kingdom

**Keywords:** cattle, dairy, mastitis, metabolic disease, diagnostic tools

## Abstract

Production diseases are highly prevalent in modern dairy herds, resulting in lost productivity and reduced animal welfare. Two important production diseases are mastitis and metabolic disorders. The availability of robust diagnostic tools that can detect animals at early stages of disease is crucial to prevent the high costs associated with lost productivity and the treatment of clinically and/or chronically diseased animals. Despite a variety of diagnostic methods being available to farmers and veterinarians, the incidence of these diseases in UK dairy herds has not changed over the last decade, underscoring the need for improved approaches for early disease detection. To this end, we administered a questionnaire to farmers and veterinarians to understand current diagnostic practices in the UK dairy cow sector, and to gather opinions on the suitability of currently available diagnostic tests in order to identify specific areas where improvement in diagnostic technologies and/or practices are needed. Data from a total of 34 farmers and 42 veterinarians were analyzed. Results indicated that most farmers surveyed used a combination of methods to diagnose mastitis and metabolic disorders, the most popular of which were visual inspection and milk recording somatic cell count data for mastitis, and body condition score and milk ketone testing for metabolic disorders. These preferences were not always in line with veterinarian recommendations of different diagnostic tools. Moreover, veterinarians indicated they were not satisfied with currently available diagnostic tools or how these were implemented by farmers. Both farmers and veterinarians recognized there was substantial room for improvement of current diagnostic tools, particularly in regard to the need to detect disease early. A majority of respondents preferred new diagnostic tests to be suitable for use with milk rather than blood or urine samples, and to yield results within 24 h. Finally, both groups surveyed identified economic cost as the most important barrier for the future uptake of new diagnostic technologies. The information obtained should guide the future development of diagnostic approaches that meet both the expectations of farmers and veterinarians, and help bring about a reduction in the incidence of production diseases in UK dairy herds.

## Introduction

**Figure 1:**
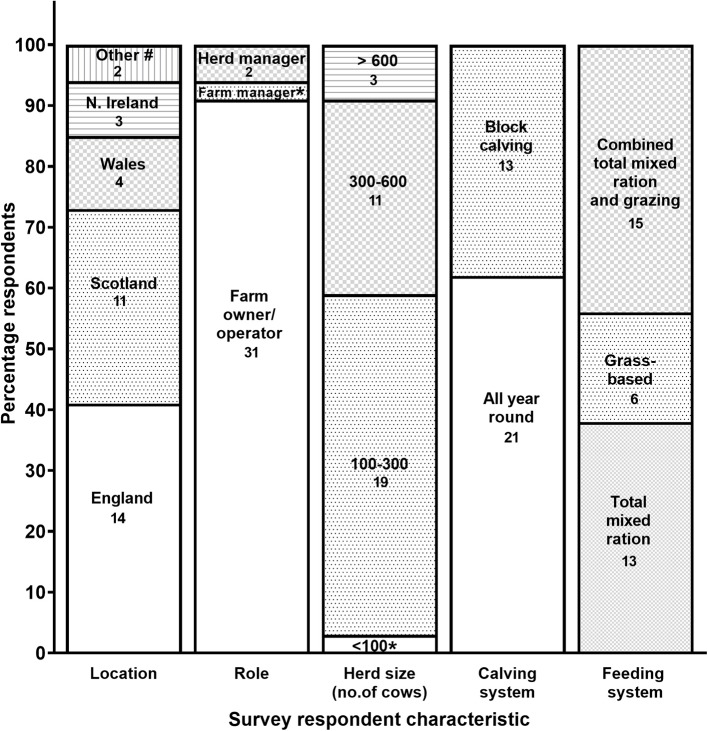
Characteristics of respondents and farms that participated in the farmer questionnaire (*n* = 34). Actual numbers of respondents for each category are also shown. ^#^Includes Guernsey and Republic of Ireland, *One respondent only.

Based on recent Agriculture and Horticulture Development Board figures (1) the UK dairy industry comprises 1.9 million dairy cows producing nearly 14 billion liters of milk every year, with a total of 13,000 active dairy farmers. Conservative estimates for the dairy industry worldwide are 300 million cows producing 600 million tons of milk every year on 120 million dairy farms. In the UK alone, milk production is worth £8.8bn at wholesale level, making up almost 20% of total agricultural output.

Keeping milk production profitable for farmers in the context of national and global economies critically depends on dairy herds maintaining good cow health. Production diseases can result from intensive dairy cow management in modern farm systems. Because of their high incidence in dairy herds, production diseases substantially limit milk production and threaten the sustainability of the dairy industry in the UK and globally (2–4). Production diseases include mastitis, infertility, lameness, and several metabolic disorders, and occur with highest frequency during the period around calving when physiological stress associated with the high energy requirements of gestation and lactation are at their greatest, thus compromising immunity and resistance to disease (5, 6). In the case of metabolic disorders (including ketosis, ruminal acidosis, hypocalcaemia, and hypomagnesaemia), although clinical disease incidence is relatively low compared to mastitis (<10 vs. 40%), subclinical cases are highly prevalent (>30%), and predispose affected cows to other production diseases as well as reducing milk production (7). In this context, the availability of robust diagnostic tools that can detect animals at early stages of disease, particularly in the case of mastitis and metabolic disease, is crucial to prevent the high costs derived from lost productivity and treatment of clinically and/or chronically diseased animals (3).

A variety of approaches are available for the early detection of mastitis and metabolic disease (8, 9). Somatic cell or bacterial counting, either in individual samples or bulk milk, or ion conductivity tests are routinely used for mastitis. For metabolic disease, body condition scoring and/or quantification of fat/protein ratios, metabolite levels (ketone bodies, fatty acids) or minerals in blood and/or milk are commonly used to establish individual or herd-wide prevalence or susceptibility to the disease. Yet the actual ability of these approaches to identify the very early stages of disease or predict likelihood of disease in healthy herds is limited, and concerns related to high cost or labor requirements may limit the uptake of some approaches. The fact that the incidence of mastitis and metabolic disease in UK herds has not changed over the last decade (3) underscores the need for novel, accurate and cost-effective methods for early disease detection. New approaches are being tested, e.g., quantification of inflammation related proteins in blood or milk (8) and composite approaches for automated systems (10), although they do not always meet the conditions allowing efficient and affordable implementation in modern farming systems. Up-to-date information on diagnostic practices and preferences by key stakeholders in dairy cow health, i.e., farmers and veterinarians, is essential to guide current and future efforts to develop successful diagnostic approaches for dairy cows.

With this in mind, we wished to gain insight into the needs of the dairy industry in relation to existing technology for the diagnosis of mastitis and metabolic disease in cows. To do this, we distributed a questionnaire among farmers and veterinarians to understand current diagnostic practices in UK dairy farms, and to reveal existing opinions on the suitability of currently available diagnostic tests and the specific areas where improvement is needed.

## Methods

Two questionnaires, one for farmers and one for veterinarians, were prepared using SurveyGizmo (11). The questionnaires were prepared using our team's combined expertise in animal science, farm animal medicine, agribusiness consultancy and dairy farming. Each questionnaire included separate questions on Mastitis diagnosis, Metabolic disorder diagnosis (including Ketosis, Hypocalcaemia, Acidosis, Fatty liver disease and Hypomagnesaemia) and Barriers to technology uptake (see Appendix in Supplementary Material). Questions were written to maximize information obtained from respondents without pre-empting/biasing their response. Restrictive settings were used that ensured each question was answered before the respondent could move onto the next question. Where multiple Likert scales were provided in succession within one question, it was ensured that a minimum of three Likert variables were answered before the respondent could progress to the next question. The two questionnaires were appropriately pre-tested “in-house” before being released online.

Online links to the farmer and veterinarian questionnaires were sent by e-mail to a total of 500 farmer and 600 veterinary contacts, respectively, maintained by the Dairy Herd Health and Productivity Service (DHHPS) at Edinburgh's Royal (Dick) School of Veterinary Studies (R(D)SVS). The DHHPS provides veterinary diagnostic and consultancy services throughout the UK. Contacts across the UK that had used DHHPA services at least once over the past 36 months were used. Moreover, questionnaires were made publicly available on twitter, requesting that only participants in the UK complete the survey. In all cases, questionnaires were available for completion online from 7th to 28th February 2019. Approval was obtained from the Human Ethical Review Committee at the R(D)SVS before the questionnaires were released.

After completion, each individual questionnaire was manually screened for any obvious signs of falsification and to ensure that the partially completed questionnaires contained information worthy of analysis (e.g., more than just demographic info). Acquired knowledge, for example of the relationship between herd sizes and various management practices, was used to assess authenticity of responses. Two questionnaires were excluded from the outset. One farmer questionnaire was excluded because the farmer was based in Kenya, and a veterinary questionnaire was excluded because the respondent was a nutritionist, not a veterinarian. Questionnaire data were analyzed as follows. SurveyGizmo was used to obtain number of responses, percentages and mean (± SE) score values, whereas 95% confidence intervals (CI) for percentages were calculated in Minitab 17 (Minitab LLC) using the One sample proportion test. All Figures were prepared using GraphPad 8.0 software.

## Results

### Respondent Demographic Information

A total of 61 out of 500 dairy farmers responded to the survey. Of those, 34/500 (6.8%) responded to all or almost all (≥70%) of the questions, and were included in the data analyses. Respondent distribution based on location, role on a farm, herd size, calving system and feeding system are shown in Figure 1. A total of 59 out of 600 veterinarians contacted undertook the questionnaire, of which 42/600 (7.0%) responded to all or almost all (≥70%) of the questions. Out of the 42 veterinarians, 23 were located in England (54.8%), 11 in Scotland (26.2%), seven in Wales (16.7%) and one in Republic of Ireland (2.4%).

### Mastitis Diagnosis

Seventeen out of 34 farmers surveyed (50.0%) reported that their average somatic cell count (cells/ml) was <150,000, and 14/34 (41.2%) reported average cell counts between 150,000 and 200,000. Only 3/34 (8.8%) of respondents reported counts above 200,000.

**Figure 2:**
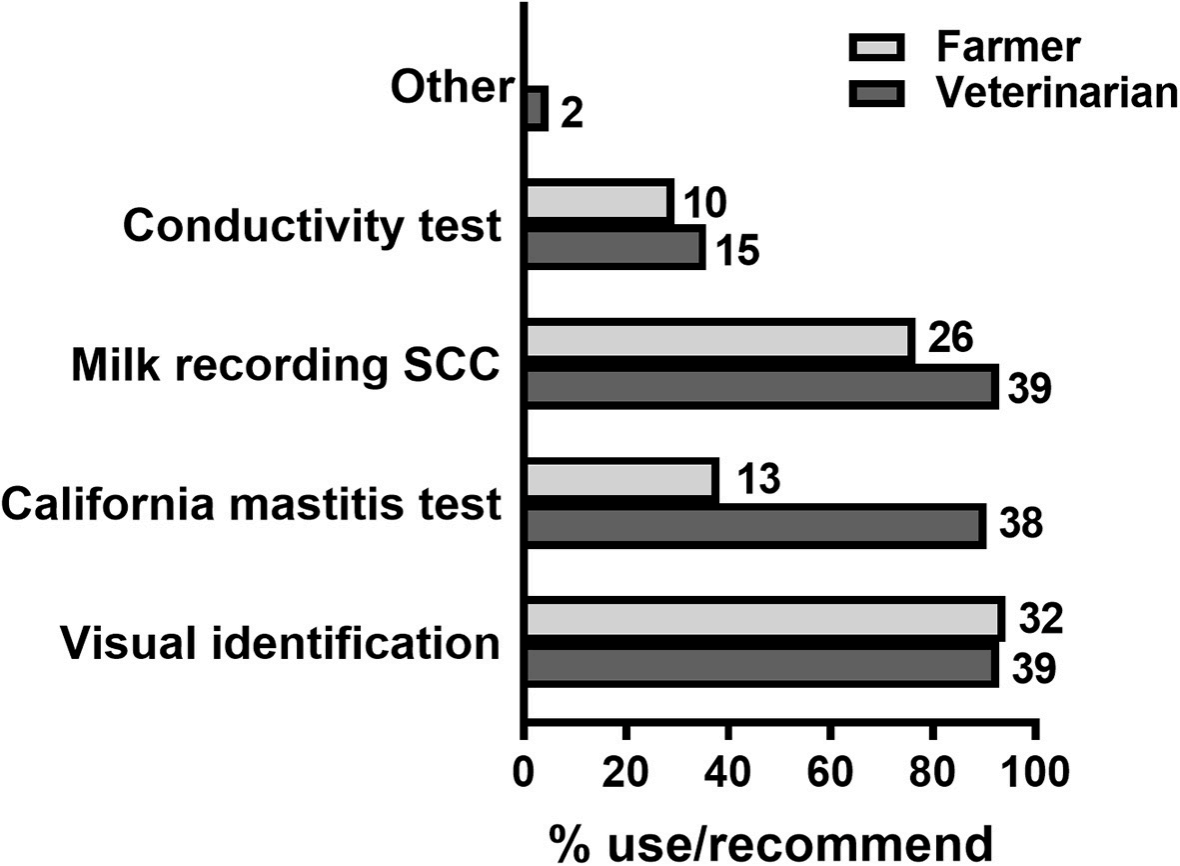
Methods and practices used for the diagnosis of mastitis. Percentages of farmers using/veterinarians recommending each method listed are shown by horizontal bars. Number of respondents are shown next to each bar. *N* = 34 farmers, 42 veterinarians.

Results of the questionnaire highlighted that 32 out of 34 farmers (94%) used visual identification to identify mastitis, usually in combination with other methods (Figure 2, Table 1). Only 4/34 farmers (12%) used a single diagnostic method (visual identification) to diagnose mastitis (Table 1). Visual identification was most commonly used with SSC data from routine individual milk recording (10/34 farmers, 29%), whereas a further 14 farmers (41%) used these two approaches together with either or both of California mastitis test (CMT) and conductivity test. Only 2/34 farmers (6%) reported not using visual identification, using instead SSC data from routine individual milk recording in combination with CMT. On the other hand, veterinarians surveyed recommended multiple methods to identify mastitis in their client's dairy cows, in particular visual identification, individual milk recording and CMT (Figure 2). When asked how often the whole herd was checked for mastitis (Table 2), 22/34 farmers (65%) responded that the whole herd was checked daily, whereas only 2/34 farmers (6%) responded that they never checked the whole herd for mastitis at one time. When queried who was responsible for identifying most cows with mastitis on farm (Table 3), questionnaire respondents identified farm workers/milk harvesters (15/34 or 44%) and herd managers (10/34 or 29%) as finding the most mastitis. Moreover, 30/34 farmers surveyed (88.2%) stated that they treated more clinical that subclinical cases of mastitis.

**Table 1:** Number of methods used to diagnose mastitis (*N* = 34 respondents).

**Number of diagnostic methods**	**No. respondents**	**% respondents**	**% respondents (95% CI)**
1	4	11.8	3.3–27.4
2	16	47.1	29.8–64.9
3	11	32.3	17.4–50.5
4	3	8.8	1.9–23.7

**Table 2:** Frequency with which the entire herd (all cows in one milking) was checked for mastitis (*N* = 34).

**Frequency**	**No. respondents**	**% respondents**	**% respondents (95% CI)**
Daily	22	64.7	39.6–72.2
Weekly	1	2.9	0.1–13.5
Monthly	4	11.8	2.9–24.2
Annually	1	2.9	0.1–13.5
When there was a high bulk somatic cell count	3	8.8	1.9–23.7
Never	2	5.9	0.7–19.7
Other	1	2.9	0.1–15.3

**Table 3:** Individual/system on farm responsible for identifying the most mastitis (*N* = 34).

**Individual/system**	**No. respondents**	**% respondents**	**% respondents (95% CI)**
Farm manager	5	14.7	4.9–31.1
Herd manager	10	29.4	15.1–47.5
Milk harvester/farm worker	15	44.1	27.2–62.1
Automated detection system	3	8.8	1.9–23.7
Other	1	2.9	0.1–15.3

**Figure 3:**
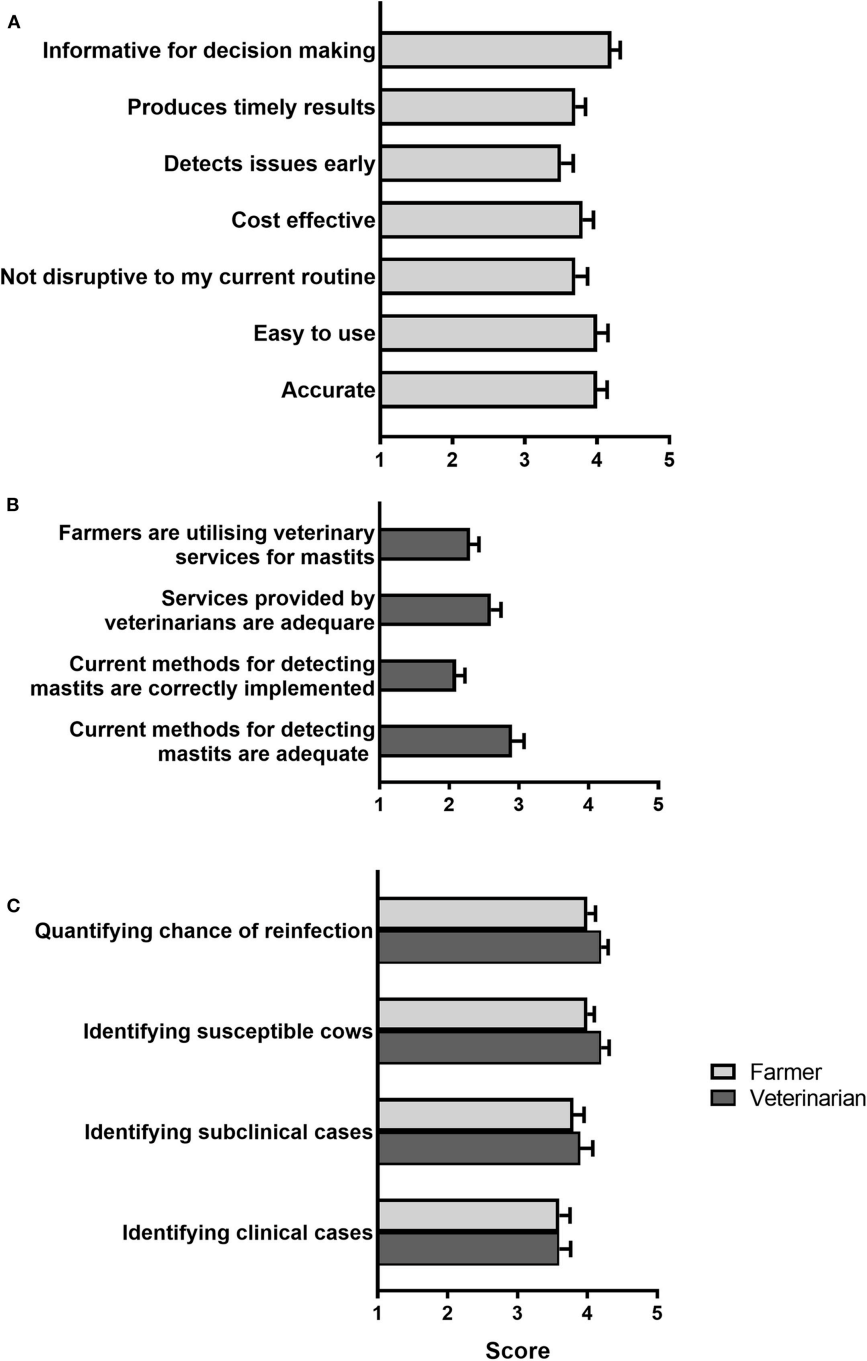
Opinions on current approaches to diagnose mastitis. **(A)** Farmer rating of the characteristics of current diagnostic methods in response to the statement “Current diagnostic tools for mastitis are…” **(B)** Veterinarian rating of different statements related to current diagnostic approaches. **(C)** Rating of the need for improvement of different aspects of current diagnostic tests. In all cases, respondents were asked to rate their agreement with each statement provided from one (strongly disagree) to five (strongly agree). Mean (± SE) scores are shown. *N* = 34 farmers, 40 veterinarians.

Farmers were then asked to rate several characteristics of current mastitis detection methods from 1 to 5 (1 = strongly disagree, 3 = neither agree/nor disagree, 5 = strongly agree; Figure 3A). Respondents agreed most (mean, 4.2 ± 0.1) with “Current tests are informative for decision making,” whereas “Current tests detect issues early” was rated lowest (mean, 3.5 ± 0.2), just above neutral. Conversely, when asked, veterinarians felt in general that the current veterinary services and methods available for detecting mastitis were inadequate and were not correctly utilized/implemented by farmers (Figure 3B). In addition, when asked to rate the need for improvement in current diagnostic methods, both veterinarians and farmers believed substantial improvement was needed particularly in the current tests' ability to identify an animal's susceptibility to mastitis, quantify the chance of reinfection and identify subclinical mastitis (Figure 3C). In regard to a test capable of identifying animals predisposed to mastitis, 41/42 veterinarians (97.6%) acknowledged they would promote such a test in order to reduce antibiotic use. Moreover, when asked if they would be willing to treat more animals for subclinical mastitis to reduce the number of clinical mastitis cases, 27/42 veterinarians (64.3%) suggested that they would be willing to promote this method compared to 15/42 (35.7%) who would be against it.

**Figure 4:**
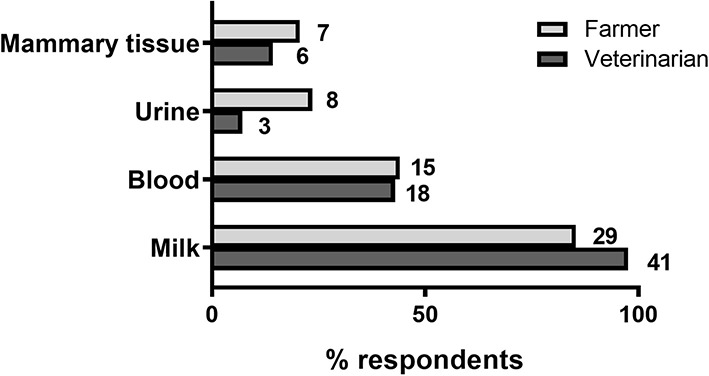
Mastitis test preferences. Percentage of farmer and veterinarian respondents indicating the suitability of each of *four* sampling sources for a mastitis test. Number of respondents are shown next to each bar. *N* = 34 farmers, 42 veterinarians.

Farmers and veterinarians were also asked about the characteristics of an ideal mastitis test. Given the choice between different sample sources for testing, farmers and veterinarians rated a milk test as the top preferred choice followed by a blood test (Figure 4). In addition, a majority of both farmers (24/34 or 71%) and veterinarians (25/42 or 60%) preferred mastitis assay results to be available within 24 h (Table 4).

**Table 4:** Turnaround time preferences for a mastitis test (*N* = 34 farmers, 42 veterinarians).

**Turnaround time**		**No. respondents**	**% respondents**	**% respondents (95% CI)**
Same day	Farmers	9	26.5	12.9–44.4
	Veterinarians	11	26.2	13.9–42.0
Overnight	Farmers	15	44.1	27.2–62.1
	Veterinarians	14	33.3	19.6–49.5
2–3 days	Farmers	9	26.5	12.9–44.4
	Veterinarians	17	40.5	25.6–56.7
5–7 days	Farmers	1	2.9	0.1–13.5
	Veterinarians	0	0	-

### Metabolic Disorder Diagnosis

Farmers and veterinarians had strikingly different perceptions of the impact of metabolic disorders on UK dairy herd health. Whereas, 40/42 veterinarians (94.7%) believed that the prevalence of metabolic disorders was a major issue on farm, only 9/34 farmers (27.3%) had the same opinion. Moreover, when asked to rank the prevalence of different metabolic disorders in UK herds, both farmers and veterinarians ranked Ketosis first, followed by Hypocalcaemia, Acidosis, Fatty liver disease and Hypomagnesaemia.

**Figure 5:**
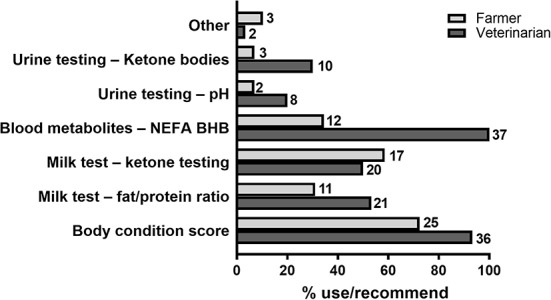
Methods and practices used for diagnosis of metabolic disorders. Percentage of farmers using/veterinarians recommending each method listed. Number of respondents are shown next to each bar. *N* = 33 farmers, 37 veterinarians.

Of the different approaches available to assess metabolic health in cows, body condition scoring was used by the largest number of farmers surveyed (25/33 or 75.8%; Figure 5). Most farmers used a combination of approaches (Table 5), with 20/33 farmers (57.6%) using body condition scoring together with one or several of the milk, blood or urine tests indicated in Figure 5, of which milk ketone analysis was the most popular as it was used by 17/33 farmers (51.5%). Two farmers (6%) indicated they used liver biopsy and daily milk yield records, respectively, as additional tests to identify metabolic disease. In addition, 34 of 36 veterinarians surveyed (94.7%) recommend the use of blood metabolites in combination with animal body condition score to identify metabolic disorders in dairy cows, and 20/36 (56%) recommended also using milk tests for ketones and fat/protein ratios for that purpose (Figure 5).

**Table 5:** Number of methods used to diagnose metabolic disorders (*N* = 33 respondents).

**Number of diagnostic methods**	**No. respondents**	**% respondents**	**% respondents (95% CI)**
1	9	27.3	13.7–46.7
2	15	45.5	29.1–65.2
3	2	6.1	0.8–20.8
4	7	21.2	9.0–38.9

When queried who was responsible for identifying most cows with metabolic disease on farm (Table 6), questionnaire respondents identified herd managers as responsible for identifying most diseased cows (15/32 or 47%) in almost half of the farms. Moreover, 19/31 farmers (61.3%) believed they detected predominantly clinical cases of metabolic disease. This figure was consistent with that obtained from surveyed veterinarians, 29/38 (76.3%) of which indicated they detected a higher proportion of clinical than subclinical metabolic disorder cases.

**Table 6:** Individual/system on farm responsible for identifying the most metabolic disorder cases (*N* = 32).

**Individual/system**	**No. respondents**	**% respondents**	**% respondents (95% CI)**
Farm manager	9	28.1	13.7–46.7
Herd manager	15	46.9	29.1–65.3
Milk harvester/farm worker	5	15.6	5.3–32.8
Automated detection system	1	3.1	0.1–16.2
Other	2	6.3	0.8–20.1

**Figure 6:**
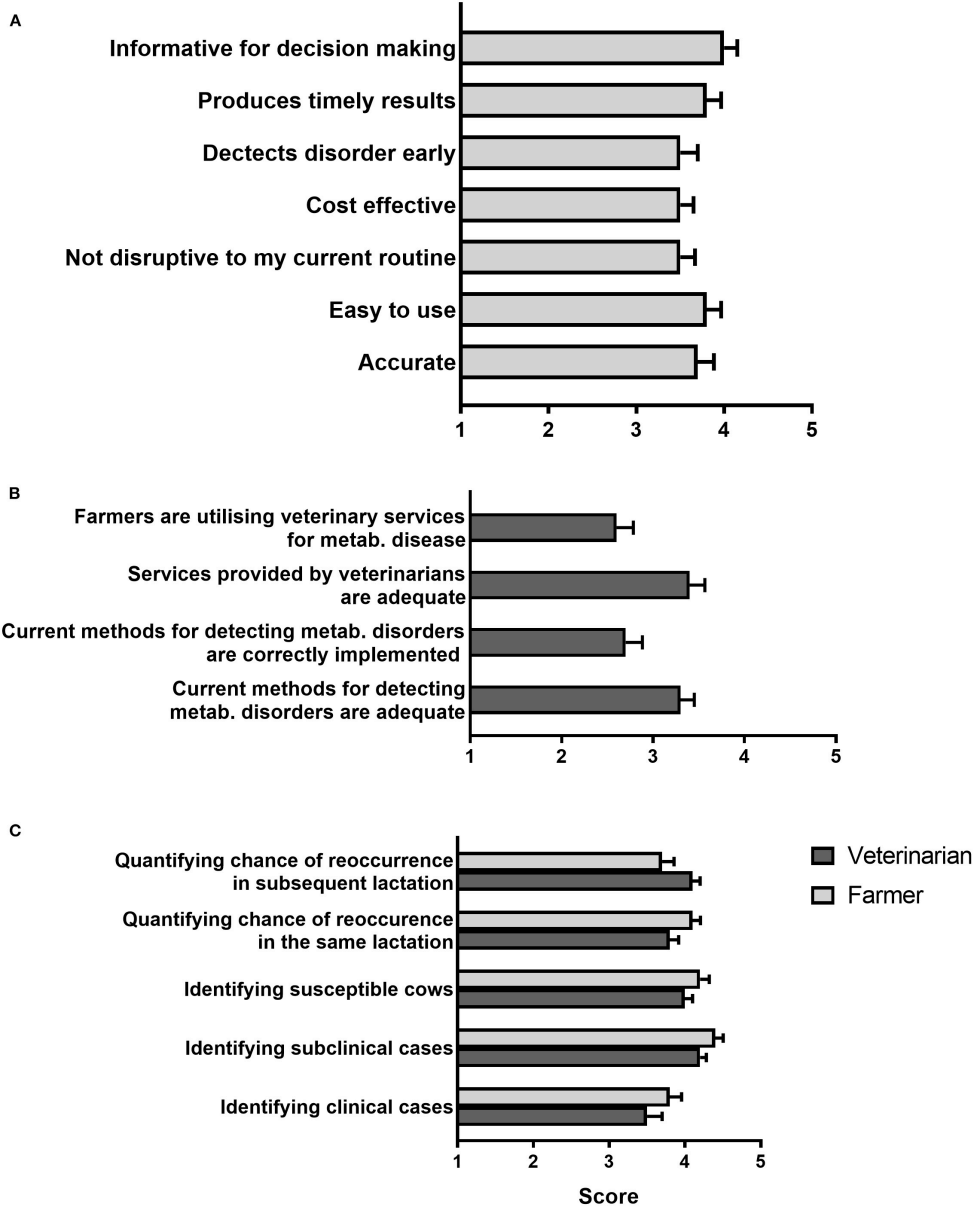
Opinions on current approaches to diagnose metabolic disease. **(A)** Farmer rating of the characteristics of current diagnostic methods in response to the statement “Current diagnostic tools for metabolic disease are…” **(B)** Veterinarian rating of different statements related to current diagnostic approaches. **(C)** Rating of the need for improvement of different aspects of current diagnostic tests. In all cases respondents were asked to rate their agreement with each statement from one (strongly disagree) to five (strongly agree). Mean (± SE) scores are shown. *N* = 33 farmers, 36 veterinarians.

Farmers rating (1–5) of different characteristics of current metabolic disease detection methods showed a somewhat positive opinion (mean score between 3.5 ± 0.2 and 4 ± 0.1 in all cases; Figure 6A). On the other hand, as was the case for mastitis tests, veterinarians believed that farmers were not fully utilizing their services and were not implementing current practices correctly on farm (Figure 6B). Indeed, both farmers and veterinarians believed substantial improvement was needed for current metabolic disease tests to identify subclinical disease, and quantify an animal's susceptibility as well as chances of disease recurrence (Figure 6C).

**Figure 7:**
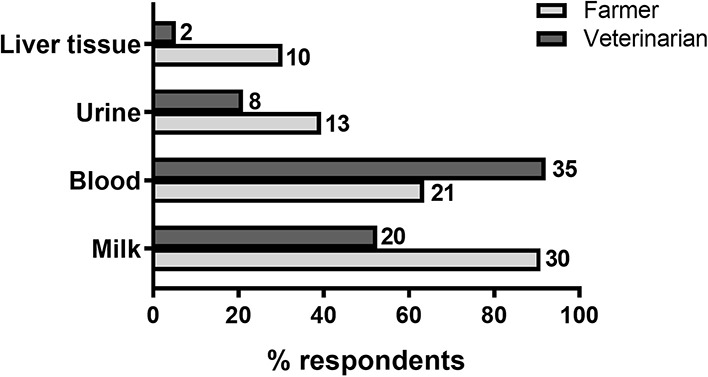
Metabolic disease test preferences. Percentages of farmer and veterinarian respondents indicating the suitability of each of *four* sampling sources for a diagnostic test. Number of respondents are shown next to each bar. *N*= 33 farmers, 38 veterinarians.

Regarding opinions on the characteristics of an ideal metabolic disease test, farmers rated a milk test as their top preference choice followed by a blood test, whereas veterinarians preferred blood to milk (Figure 7). In addition, just over half of farmers (20/33 or 61%) and veterinarians (25/38 or 66%) would prefer metabolic disease assay results to be available within 24 h (Table 7).

**Table 7:** Turnaround time preferences for a metabolic disease test (*N* = 33 farmers, 38 veterinarians).

**Turnaround time**		**No. respondents**	**% respondents**	**% respondents (95% CI)**
Same day	Farmers	9	27.3	13.3–45.5
	Veterinarians	15	39.5	24.0–56.6
Overnight	Farmers	11	33.3	18.0–51.8
	Veterinarians	10	26.3	13.4–43.1
2–3 days	Farmers	12	36.4	20.4–54.9
	Veterinarians	12	31.6	17.5–48.7
5–7 days	Farmers	1	3.0	0.1–15.7
	Veterinarians	1	2.6	0.1–13.8

### Barriers to Technology Adoption

**Figure 8:**
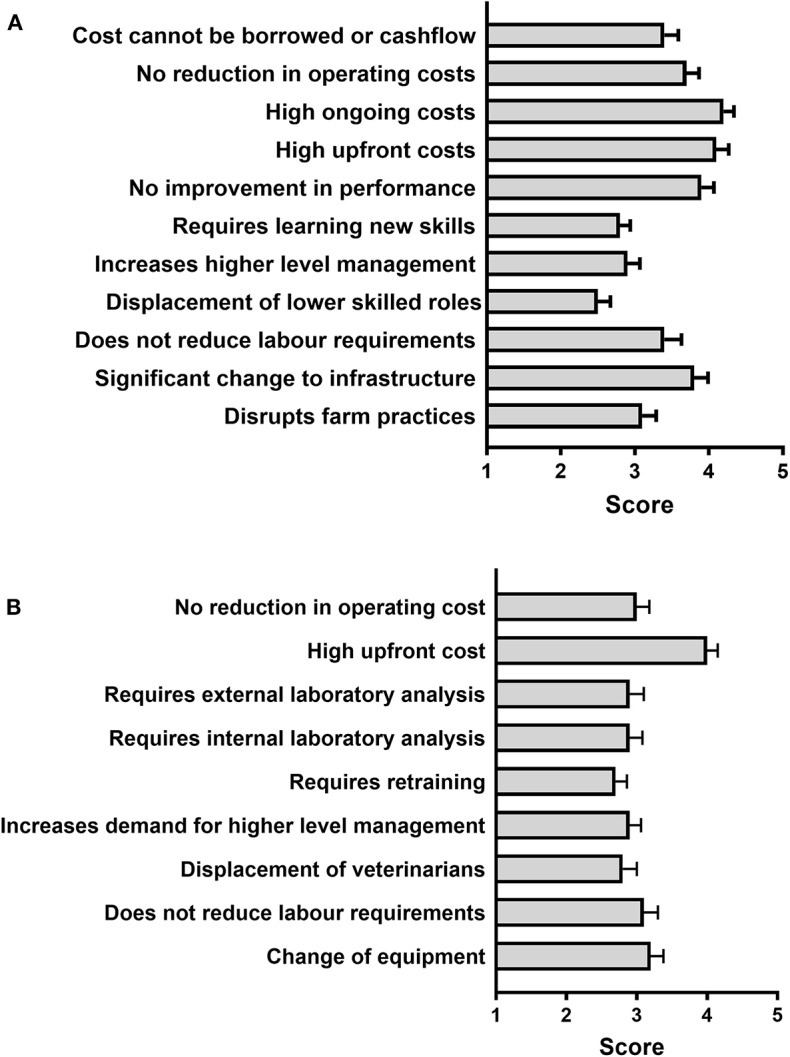
Farmer **(A)** and Veterinarian **(B)** rating of different potential barriers to new technology uptake in their farms/practices. Respondents were asked to rate each statement given from one (no barrier to adoption) to five (major barrier to adoption). Mean (± SE) scores are shown. *N* =33 farmers, 37 veterinarians.

When asked to rank different potential barriers to the uptake of new diagnostic technologies (Figure 8A), farmers identified high upfront costs and high ongoing costs as the biggest barriers. No improvement in performance, need for significant changes to infrastructure and no reduction in operating costs were also important factors. All other factors rated closer to neutral (mean score between 2.5 ± 0.2 and 3.4 ± 0.2) on the 1 (no barrier) to 5 (major barrier) scale, with displacement of lower skilled roles and the requirement to learn new skills rated as the smallest barriers to overcome.

In comparison, veterinarians regarded high upfront cost as the only major barrier (median score = 4.0 ± 0.1) to the uptake of new technology in their practices, with all other potential barriers rated closer to neutral, as shown in Figure 8B.

## Discussion

This study provided new information and opinions on current dairy herd diagnostic practices in the UK. Respondents were primarily selected from an updated UK-wide list of farmer and veterinarian users at the R(D)SVS dairy herd health services, that is representative of each of the two professional sectors in the UK. The percentages of farmer and veterinary contacts that actually completed each survey (about 7% each, see Results section) were slightly below the response rate (10–15%) typically expected with this type of surveys (www.surveygizmo.com), providing a margin of error (90%) of 12.3 and 13.8%, respectively. Moreover, respondent profiles in terms of herd size, calving and feeding systems, and geographical distribution (Figure 1) were representative of the wider UK dairy industry (1). Participants were self-selected volunteers who actively use information technologies (as these were online questionnaires). Voluntary respondents in a survey typically tend to be members of the sample populations (UK farmer and veterinary communities in this case) that are more concerned about the topic under survey and have also stronger opinions about it (12). Consequently, these groups are expected to be more willing to implement changes, or at least consider doing so, in order to improve dairy husbandry and health practices and profitability, as well as more likely to adopt new diagnostic practices and technology and, in the case of veterinarians, to recommend them to their clients. The above limitations, including potential biases, should be taken into account when interpreting the results of this study and implementing the suggested recommendations.

The combined results of the two questionnaires clearly indicated that in general surveyed farmers do not make full use of available diagnostic approaches for mastitis and metabolic disease, and in addition highlight a need for improved diagnostic tools that can better identify animals at early stages of disease. Addressing these two aspects will be key to successful implementation of early intervention strategies that can effectively reduce the current incidence of clinical disease and associated production losses incurred by dairy farmers.

In relation to mastitis, of the four diagnostic procedures considered, only CMT and conductivity tests, when used routinely on farm, may allow for prompt detection of pre-clinical disease, enabling effective reduction of clinical mastitis cases through early intervention measures (8). Yet just above 2/3 of farmers surveyed (23/34 or 68%) use either of these two techniques for diagnosing mastitis on farm, while most (26/34 or 76%) use SCC from milk recording data. The relatively low uptake of CMT, a simple and low-cost approach that can be used independently of automated milking systems, is in contrast with the high percentage of veterinarians that recommend it. Thus, encouraging wider use of CMT by farmers may in general be effective itself in reducing the incidence of mastitis in UK farms.

Similar to mastitis, most farmers surveyed (24/33 or 73%) use a combination of diagnostic approaches to assess metabolic status in their cows. In the majority of those cases (26/33 or 79.2%), these include body condition scoring and metabolite analyses in milk or blood. In contrast, only a small proportion of farmers (5/33 or 15.2%) favored the use of urine samples for diagnostic testing, in agreement with veterinarian preferences. Quantification of blood metabolites is considered the gold standard for the diagnosis of hyperketonaemia, and available blood-based tests have shown to have higher accuracy than cow-side tests using milk or urine (9, 13). Yet despite blood metabolite testing being the most widely recommended of all diagnostic approaches (100% of veterinarians surveyed), only 12/33 farmers (36.4%) indicated they routinely use this approach for diagnosing metabolic disease, instead being more in favor of milk sample testing. Thus, assay simplicity and low cost, as is the case for cow-side milk-based assays, is a primary determinant of farmers' choice for a diagnostic test for their herd.

Although farmers had a moderately positive opinion of current tests for the diagnosis of mastitis and metabolic disorders, they also believed there is substantial room for improvement, especially in regards to the ability of available tools to detect disease early. This is in agreement with the reduced number of subclinical cases compared to clinical cases detected in the surveyed farms, as shown by a majority of respondents stating that they predominantly treat clinical over subclinical cases for both mastitis (88.2% of farmers) and metabolic disease (61.3% of farmers, see Results section). On the other hand, in general veterinarians did not believe that tools and veterinary services currently available for the diagnosis of metabolic disorders, and especially mastitis, are adequate, including the limited ability of available tools to detect subclinical disease, in agreement with farmers' opinions. Importantly, veterinarians were also concerned that farmers do not make full use of veterinary services and that current diagnostic methods are not appropriately implemented on farm. Based on these opinions, a need for tests that are farmer-friendly (preferably cow-side using milk samples) and able to identify animals at early disease stages and/or at risk of disease should guide future research and test development efforts in dairy cow diagnostics. In addition, as would be expected, economic concerns including implementation and running costs, as well as cost effectiveness in the context of farm operations, topped the list of factors seen by farmers as potentially limiting the uptake of new diagnostic technology. From the point of view of the animal health diagnostics sector, the development of commercially-viable kits using new technologies that meet all farmer's requirements, particularly cost expectations, will be a challenge.

Questionnaire results highlighted several discrepancies between farmer and veterinarian opinions, specifically in relation to the impact of metabolic disorders on herd health and productivity, and the recommended vs. actual use of specific diagnostic tools for mastitis and metabolic disorders. Based on this finding, appropriate farmer education on the benefits and advantages of the different diagnostic approaches available would facilitate decision-making by farmers based on solid clinical evidence. This is essential to bring about an effective reduction of the incidence of clinical mastitis and metabolic disease in UK farms, and prevent major industry losses in terms of milk production and animal welfare.

In summary, this study highlighted current diagnostic practices related to dairy herd mastitis and metabolic disorders in the UK farms surveyed. Responses from the farmers and veterinarians surveyed revealed major gaps in both available technology and its application on-farm to effectively diagnose disease in cows. The results indicate a need for new and/or improved diagnostic tools able to accurately detect disease early, whilst at the same time being farmer-friendly (e.g., suitable for use in milk) and affordable. Together with appropriate farmer education on the importance of early diagnosis and the best approaches available, this information should guide the development of diagnostic kits that meet both the expectations of farmers and veterinarians, and assist in bringing about a reduction in the incidence of production disease in UK dairy herds.

## Data Availability Statement

All datasets generated for this study are included in the article/Supplementary Material.

## Ethics Statement

Approval was obtained from RDSVS Human Ethical Review Committee.

## Author Contributions

FD, NH, MH, TB, and AM designed the study. NH and MB carried our the survey. FD, NH, and CE analyzed the survey data. FD, NH, CE, and AM wrote the manuscript. All authors approved the final manuscript.

### Conflict of Interest

NH, MH, and TB are employed by AbacusBio International Ltd. The remaining authors declare that the research was conducted in the absence of any commercial or financial relationships that could be construed as a potential conflict of interest.
